# Refining Capture and Collaring Protocols for Red Foxes

**DOI:** 10.1002/ece3.72656

**Published:** 2026-01-19

**Authors:** Holly M. English, Patricia Romero, Lorraine Bull, Barry Nolan, Paolo Bongi, Vilhelmiina Huuskonen, Simone Ciuti

**Affiliations:** ^1^ School of Biology and Environmental Science University College Dublin Dublin Ireland; ^2^ UCD Veterinary Hospital, School of Veterinary Medicine University College Dublin Dublin Ireland; ^3^ Biodiversity Officer, Parks, Biodiversity and Landscape Services Dublin City Council Dublin Ireland; ^4^ Integrated Wildlife Management Services Laois Ireland; ^5^ Hunting Office, Ambito Territoriale di Caccia – Massa Aulla Italy

**Keywords:** movement ecology, red fox, tagging, trapping, urban wildlife, *Vulpes vulpes*, wildlife welfare

## Abstract

Wildlife species are often captured in ecological studies to take morphometric measurements, collect biological samples and/or fit animal‐attached tags to collect data on movement and behaviour. Capture may be difficult depending on the target species, with implications for the effort required by field teams, overall capture success and study goals. Though routine practice, wildlife captures have important welfare implications which should be carefully considered prior to each study. Full details on capture protocols are rarely shared with the international community, often limited to short descriptions in the methods sections of papers. More detailed information sharing can improve knowledge on methods that lead to increased or reduced capture success, saving researcher time and resources and, most importantly, boosting animal welfare. Here, we share detailed capture protocols for improving trapping success and optimising welfare protocols for our trap‐shy target species, the red fox ( *Vulpes vulpes* ). We report on the methodological refinements used to successfully trap urban red foxes in Dublin, Ireland (*n* = 16 captures), as well as related efforts as part of a pilot study in rural Tuscany, Italy (*n* = 3). We recommend setting multiple capture sites but caution against prolonged time spent in traps for foxes. Remote transmission camera traps and remote trap alert systems are highly recommended, wherever possible, to remotely monitor multiple trapping sites at once with reduced disturbance and to facilitate quick arrival at capture sites. We discuss a cheap, lightweight collar drop‐off solution to negate the need for a second capture for collar retrieval. In Dublin, we found the likelihood of capturing a fox was significantly affected by rainfall but not temperature. We conclude with an easy‐to‐consult checklist, providing advice on trap setting, pre‐baiting, collar drop‐offs and weather conditions to aid researchers embarking on the capture of foxes and other difficult‐to‐trap species, particularly in urban areas with high levels of human activity.

## Graphical abstract

Animal capture is an important aspect of ecological studies, required for fitting tags and collecting biological samples. Capture success can be increased while ensuring high welfare standards through careful consideration of how many trapping sites to use and remote trap monitoring, for example.

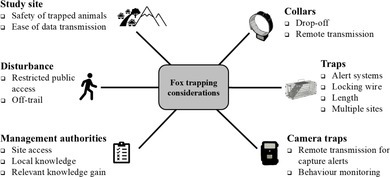

## 1 Introduction

Despite the prevalence and fundamental importance of trapping in ecological research, detailed capture protocols and related animal welfare considerations are frequently not included in published outputs (Wilson and McMahon 2006; Iossa et al. 2007). Trapping protocols are often only briefly mentioned in the methods sections of scientific publications, with insufficient information provided to ensure replication by other researchers and ensure animal welfare standards are met (Caravaggi et al. 2021). Omitting these details results in an inability for researchers to replicate trapping protocols without contacting the original researchers directly, which is not always possible and with the relevant insights remaining obscure to a broader audience (Haddaway and Verhoeven 2015). It is therefore of fundamental importance to share methodological details and advances gained in developing trapping protocols so that (i) researchers can learn from others' mistakes and (ii) researchers can reduce time, effort and resources wasted in repeating protocols which have been previously trialled unsuccessfully but remained unreported (Jakob and Long 2016). Sharing such information allows for more informed, reliable capture protocols with positive outcomes for animal welfare and conserved researcher effort.

Capture protocols should also consider variation between individuals, populations and species (Tuyttens et al. 1999; Stokes 2012). Inter‐individual variation, or personality, may result in more explorative, risk‐taking individuals being captured more often than less explorative, risk‐averse conspecifics (Biro and Dingemanse 2009). At the population level, there may be differences in trappability driven by the degree of familiarity with human activity or past interactions with humans, such as that experienced between urban and rural populations (Shivik et al. 2005; Stillfried et al. 2017). Urban wildlife are more familiar with human structures and activities, which may make novel objects such as traps appear less daunting (Shivik et al. 2005; Barrett et al. 2019; Jarjour et al. 2020). Conversely, urban wildlife can maintain high levels of neophobia to avoid negative interactions with humans (Feng and Himsworth 2014; Mazza et al. 2021), leading to heightened caution around traps. The lack of consensus regarding neophobia versus neophilia in urban populations (Griffin et al. 2017) suggests that species‐specific investigations are warranted, with these traits having consequences for trapping and monitoring efforts.

Indeed, there is considerable variation in how easy or difficult it can be to capture a particular species, with consequences for fieldwork protocols, researcher effort and animal welfare considerations. The red fox ( *Vulpes vulpes* ) is known to be difficult to capture for ecological studies (Kay et al. 2000), despite being one of the most studied carnivore species globally (Brooke et al. 2014) and having the largest range of any wild extant member of the carnivore family (Sillero‐Zubiri and Macdonald 2004). Despite the considerable body of research already conducted on the red fox, this species is of continued research interest for many reasons. Foxes show variable behavioural traits across their large range (Cavallini 1996; Sillero‐Zubiri and Macdonald 2004; Main et al. 2020), come into conflict with humans in rural and urban areas (Kimmig et al. 2020; Basak et al. 2022), are sometimes managed in response to conservation efforts for other species (Fletcher et al. 2010; Tobajas et al. 2020) and are controlled as an invasive species in parts of their range (Harding et al. 2001; Mahon 2009). Due to this wealth of research, foxes may be considered an important ecological model organism. The extensive research conducted to date on the red fox, its wide range, cosmopolitan distribution and common occurrence in human‐dominated environments make it a suitable candidate for long‐term studies on adaptation to global change, human‐wildlife conflict and coexistence, invasive species ecology and predator control.

Another important consideration in wildlife capture is that efforts to boost capture success do not lead to sub‐optimal welfare standards for captured animals. Many studies have reported higher capture success rates using capture devices that restrain a single leg (such as cable restraint devices or padded foot snares) than with box or cage traps, which contain an animal as a door is triggered to close upon stepping on a treadle (Shivik et al. 2005; Muñoz‐Igualada et al. 2008; McCarthy et al. 2013). Leg‐restraining traps have also frequently been reported to have higher injury rates; however, particularly to juveniles or non‐target species that may also be captured (Muñoz‐Igualada et al. 2008; McCarthy et al. 2013). The frequency of trap checks is another important aspect of trapping with relevance to both capture success and animal welfare. Reducing the frequency of trap checks can reduce effort and improve capture rates of species wary of human scent and activity (Arthur 1988; Benevides Jr. et al. 2008), but may risk captured animals spending longer periods in traps (McCarthy et al. 2013). Longer time periods spent in traps are often associated with more serious injuries, higher exertion and increased rates of stress behaviours (Iossa et al. 2007; Proulx 2022). Reported trap check times for red foxes vary, with different studies typically reporting checks every 12 h (Kobryn et al. 2023) or every 24 h (Muñoz‐Igualada et al. 2008) and many studies not reporting the frequency of trap checks. Reducing time spent in traps, for example through remote trap monitoring systems, is recommended to improve the welfare of captured animals (Iossa et al. 2007; McCarthy et al. 2013; Keiter et al. 2022). Potential welfare refinements to trapping protocols are an important area of research, particularly for species such as foxes that are widely captured for research and management.

**FIGURE 1:**
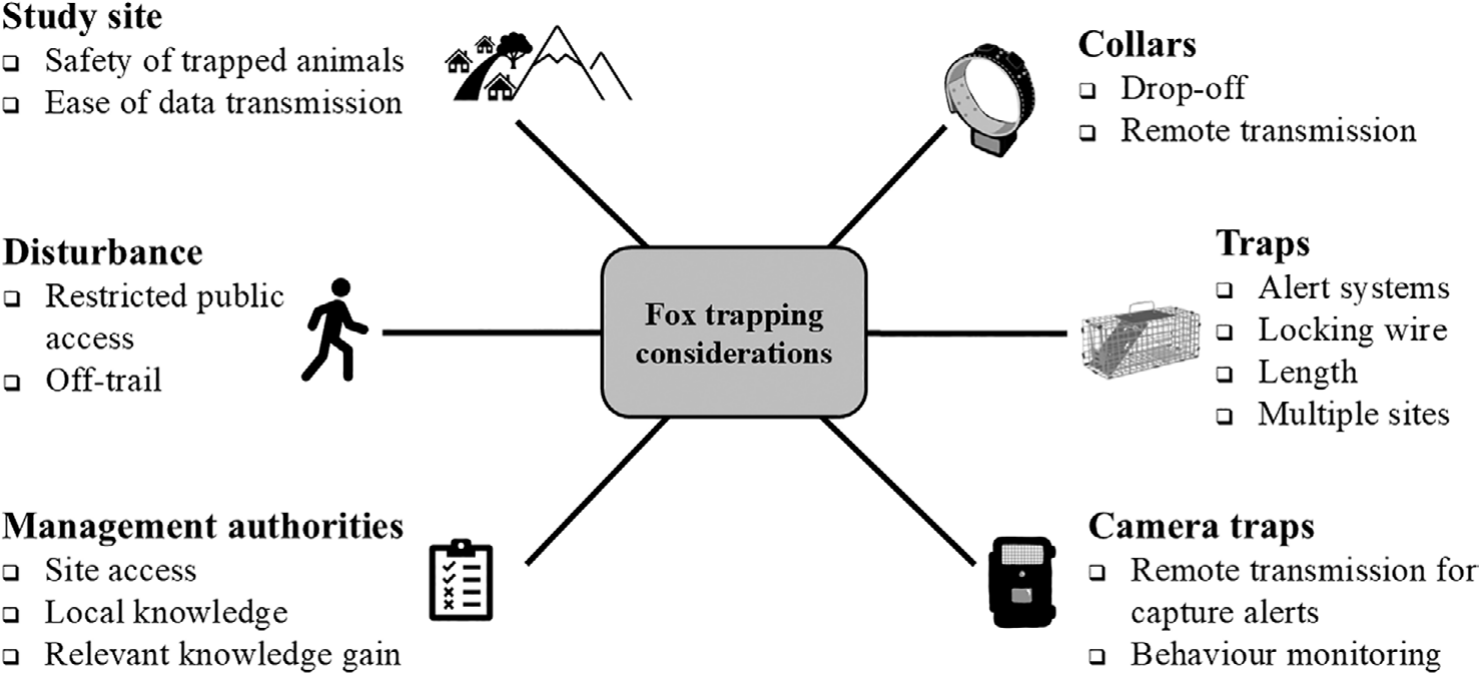
Infographic showing key considerations for refining capture protocols for both rural and urban foxes. This project arose through collaboration with local management authorities, facilitating suitable site selection and access with minimal disturbance. Working with local management authorities was also considered important for local knowledge and ensuring research outcomes were in line with management goals. Specific features of collars, traps and camera traps were trialled in efforts to improve capture success, maintain high animal welfare standards and boost data recovery. GPS collar, trap and camera trap silhouettes created by Gabriela Palomo‐Munoz.

Documenting issues encountered and resolved can aid other researchers working on trap‐shy species to improve capture success while maintaining high welfare standards. Here we present challenges encountered and subsequent methodological refinements in capturing red foxes as part of a collaborative project on movement ecology and wildlife anaesthesia protocols. We report on capture efforts on urban foxes in Dublin, Ireland, taking place from 2022 to 2024, following a related pilot study on rural foxes in Tuscany, Italy, in 2022. We present detailed considerations and refinements implemented to test whether they increased the capture success of a trap‐shy species (Figure 1). Specifically, we:

- Partnered with management authorities to select study sites in areas with limited disturbance.
- Trialled collars with custom drop‐off mechanisms and remote transmission capabilities to reduce the need for recaptures and boost data retrieval.
- Deployed camera traps to assess fox behaviour around traps.
- Adjusted trap length and added a locking trigger mechanism based on camera trap footage.
- Fitted traps with alert systems, i.e., trap alert tags and remote camera traps, to notify the field team of captures.
- Explored trapping success in relation to weather conditions, specifically temperature and rainfall.

## 2 Materials and Methods

Data were collected in two distinct environments representing distinct management goals. We collaborated with two management authorities—a city council in Dublin (Ireland) and a game management authority in Lunigiana (Tuscany, Italy).

### 2.1 Ethics Statement

All fox captures and related activities were carried out with full ethics approval. In Dublin, the study was approved under AREC‐22‐10‐Huuskonen by University College Dublin at the university level and AE18982/P216 by the Health Products Regulatory Authority (HPRA) at the national level. In Lunigiana, approval was granted under AREC‐E‐21‐61‐Ciuti by University College Dublin and 89/2022 by Direzione Agricoltura e Sviluppo Rurale (Regione Toscana).

### 2.2 Selecting Trapping Locations

**FIGURE 2:**
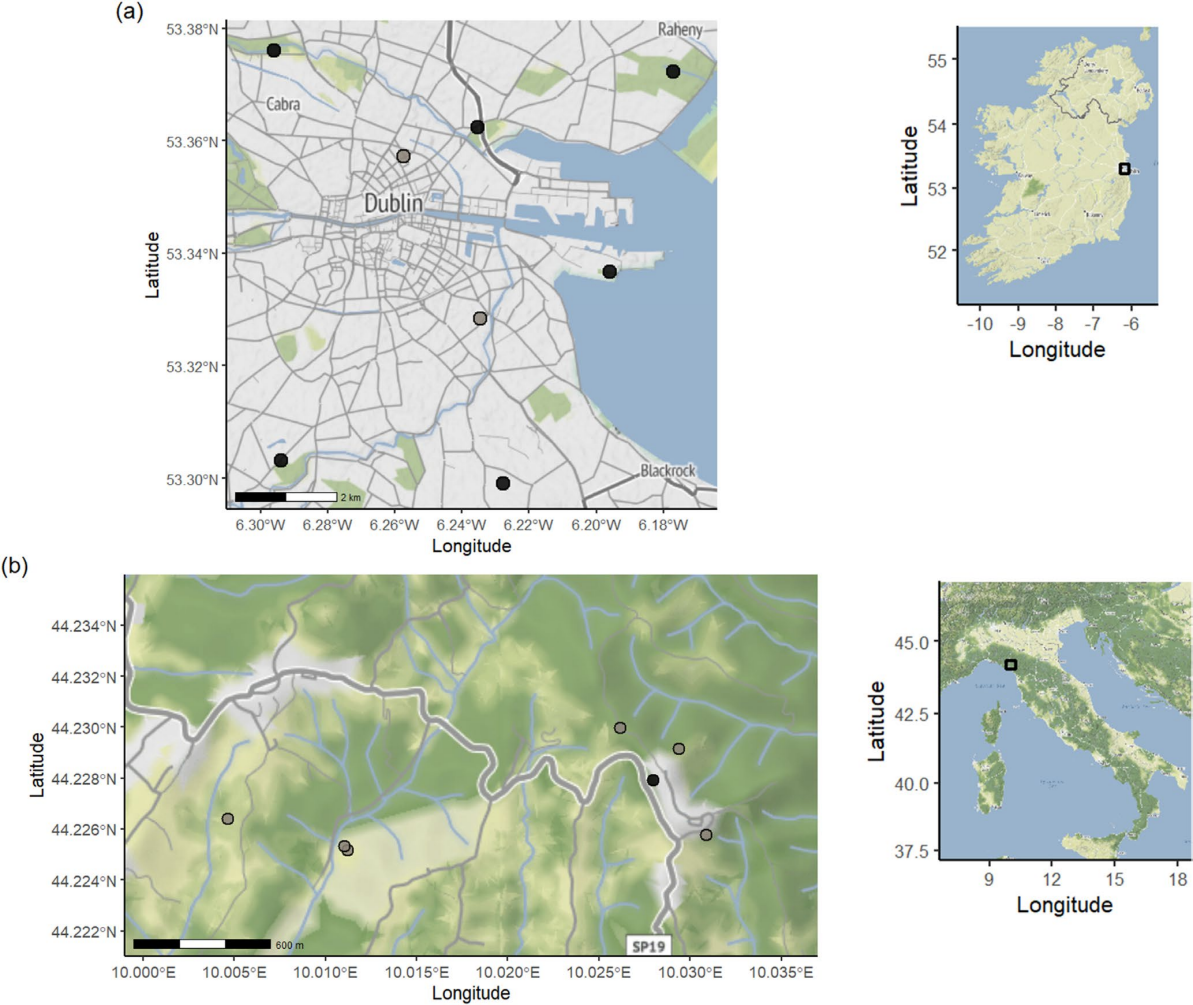
Trapping sites in (a) Dublin and (b) Lunigiana. Black circles indicate sites with capture success while grey circles show sites where trapping efforts were conducted but no foxes were captured. The inset shows the location of the study sites within Ireland and Italy, respectively.

In Dublin, trapping sites were spread across the city so that different conditions within the urban environment were represented (Figure 2a). Trapping locations were chosen through close consultation with Dublin City Council's Biodiversity Officer and park management staff. Traps were placed in secure locations to prevent disturbance or interference from members of the public (Figure 1). Five trapping sites were in gated park depot areas, one was in a pitch‐and‐putt golf course which was locked overnight, one was in a secured wildlife management area in an industrial estate and one was in the garden of a private, residential property (Table 1).

**TABLE 1:** Metadata of animals captured across all trapping locations, listed in order of date of capture.

Year	Fox ID	Sex	Region	Site	Site type
2022	Samantha	F	Lunigiana	Olivola	Village edge
	Megan	F	Lunigiana	Olivola	Village edge
	Samantha (R)	F	Lunigiana	Olivola	Village edge
2023	Ruby	F	Dublin	Bushy Park	Gated park depot
	Rocky	M	Dublin	Bushy Park	Gated park depot
	Freddie	M	Dublin	Bushy Park	Gated park depot
	Fantastic Mr	M	Dublin	St. Anne's Park	Gated park depot
	Merida	F	Dublin	Tolka Valley Park	Pitch and putt course
	Eevee	F	Dublin	Bushy Park	Gated park depot
	Tom	M	Dublin	Irishtown Nature Reserve	Wildlife management area
	Alfie	M	Dublin	Clonskeagh	Private garden
2024	Kas	F	Dublin	Bushy Park	Gated park depot
	Daría	F	Dublin	Fairview Park	Gated park depot
	Ash	M	Dublin	Bushy Park	Gated park depot
	Gráinne	F	Dublin	Fairview Park	Gated park depot
	Freddie (R)	M	Dublin	Bushy Park	Gated park depot
	Tobi	M	Dublin	Fairview Park	Gated park depot
	Rua	F	Dublin	Fairview Park	Gated park depot
	Ash (R)^a^	M	Dublin	Bushy Park	Gated park depot

*Note:* A capital R indicates recaptures.

^a^ Released without manipulation.

In Lunigiana, traps were placed in rural sites that did not have restricted public access (Figure 2b). To minimise disturbance in this setting, traps were placed in zones where human hunting activities were not allowed (which was also a requirement of the trapping permit). Care was taken to ensure traps were not visible from roads or hiking trails (Figure 1).

### 2.3 Trapping Protocols and Trap Specifications

**FIGURE 3:**
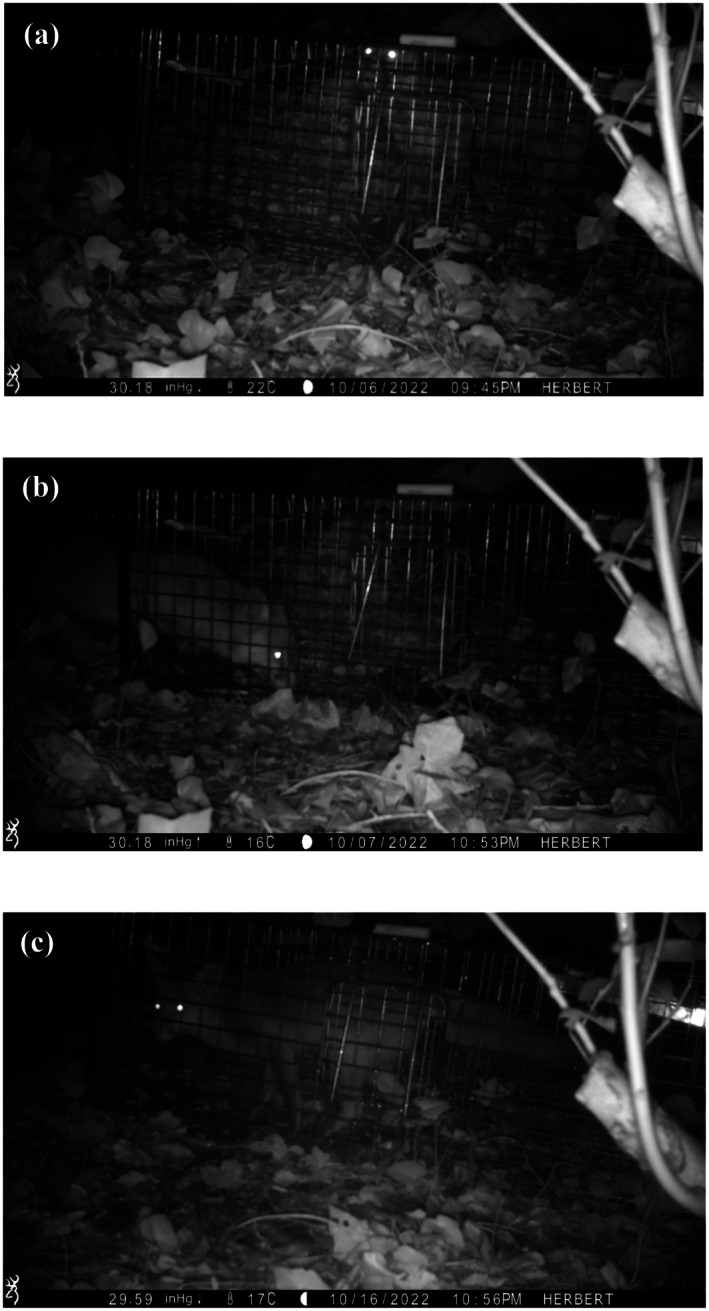
These three camera trap video stills show stages of trap investigation during initial pre‐baiting: (a) Inspecting the outside of the trap, including sniffing and circling. (b) Placing the head and forelegs inside the trap before retreating. (c) Full body inside the trap and bait taken.

In Dublin, trapping sites were pre‐baited for approximately 1 week before trapping efforts began. The traps were left open and unarmed and baited daily to allow foxes to become familiar with the traps and associate them with food. A camera trap (Browning, USA or Victure, UK), which in this preliminary stage did not have remote transmission capabilities, was mounted nearby with the trap in view. Camera traps allowed us to determine when foxes were readily entering traps so that active trapping attempts could commence (Figure 3). Initial capture attempts took place in Autumn 2022, with one of two trapping sites, a private garden, being intensely monitored each night with hourly trap checks without disturbance from a window. The other site, a gated park depot, required a member of the field team to approach the trap for trap checks. A single trap was deployed and set in each of these trapping sites. Camera traps were deployed in both sites to inspect fox behaviour in between hourly visual trap checks.

**FIGURE 4:**
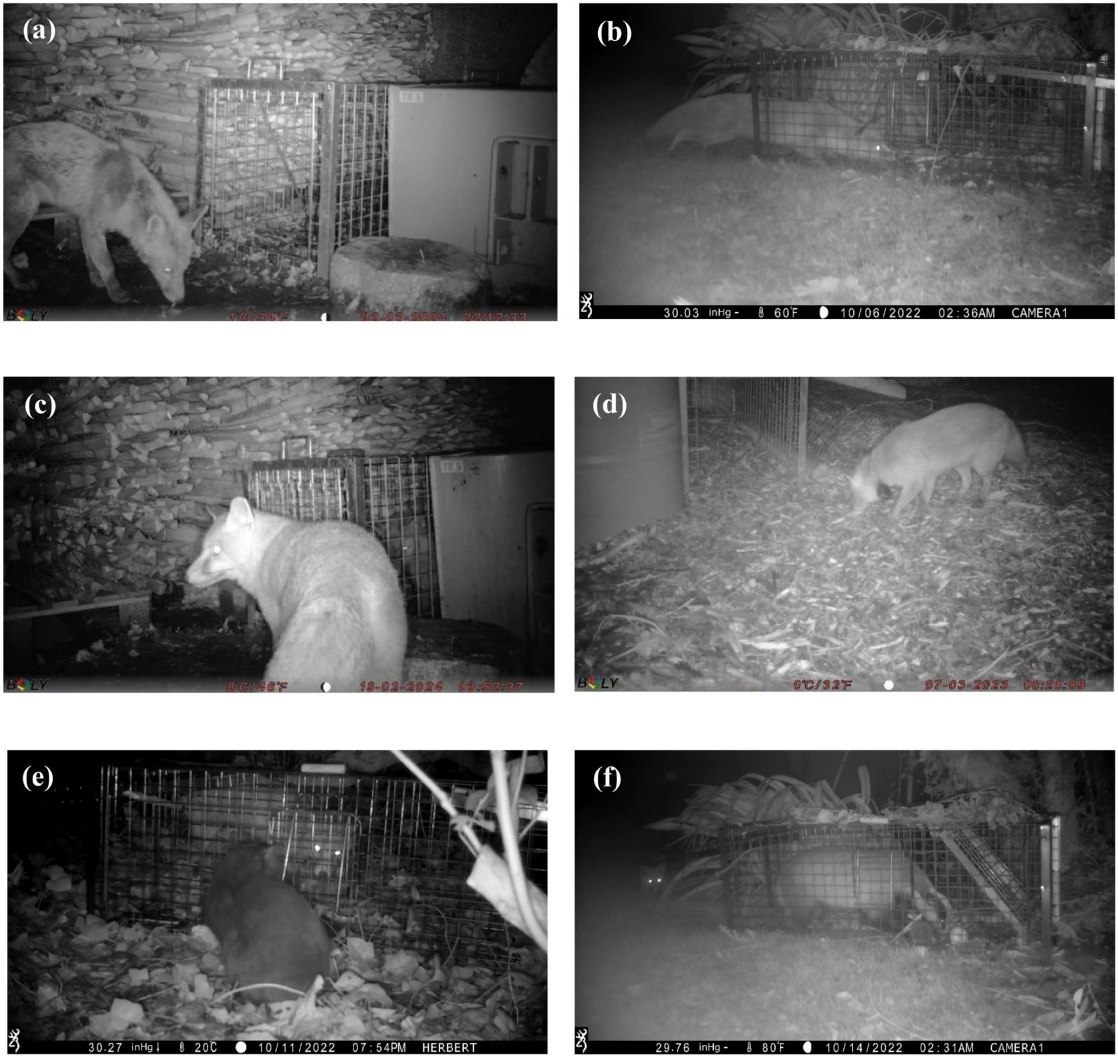
Camera trap photos or video stills illustrating additional behavioural insights that were gained from camera trapping. (a) Camera traps confirmed whether foxes were visiting traps, which was important both before commencing the trapping attempt and choosing whether to continue using a particular trapping site during ongoing trapping efforts. (b) In shorter, two‐door traps, foxes were able to reach centrally placed bait by stretching and pushing back against closing trap doors, evading capture. (c) A second fox outside the trap containing a captured fox. Observing trapped individuals may have educated other foxes in the area about the potential to become trapped. (d) Revisits by collared foxes indicated continued use of the trapping sites following capture, which may vary between individuals. Regular interspecific interactions were documented, with camera trap videos frequently capturing foxes visiting traps with (e) cats or (f) badgers present, without apparent direct aggression.

Following consistent failure to trap foxes, efforts were scaled up so that 3–4 trapping sites were active on a given night in Dublin, with 1–3 traps set per site. An independent wildlife management consultant was contracted to offer guidance and assist with trapping efforts. Trap alert tags (Perdix, UK) and remote transmission camera traps (Perdix, UK) were deployed to minimise disturbance to the trapping site from manual trap checks, which were thought to contribute to the unsuccessful pilot Autumn capture season. Remote monitoring allowed us to reduce disturbance while continuing to minimise the amount of time captured foxes spent in traps. Sites with captures were prioritised and travelled to before sites with no captures, and the remote cameras further provided immediate information about the length of time the fox had spent in the trap and its behavioural state (Figure 4).

The cage traps used varied in measurement. The initial Autumn 2022 capture attempts in Dublin used Alive Predator Cage Traps (Tirlán Farm Life, Ireland). Traps measured 40 × 33 × 120 cm and featured a door on each end with a central treadle that triggered both doors to close when pressed. A two‐door trap was used following anecdotal advice received concerning how foxes react as a trap triggers. Following unsuccessful trapping attempts, it was deemed that the length between the trap doors and central treadle was not long enough and different traps should be used (Figure 4b). During the 2023 and 2024 Spring captures, traps measured 52 × 46 × 153 cm. These traps had one door, and bait was placed at the opposite end. Additionally, these traps were reinforced with a wire locking mechanism, which fell to lie against the trap door. The locking mechanism prevented captured foxes from pushing the door up before it had fully shut upon the trap triggering, as had been witnessed from camera trap footage during the Autumn trial (Figure 4b).

In Lunigiana, cage traps were also used, each with two doors and measuring 38 × 38 × 116 cm. While the two‐door traps initially trialled in Dublin had fixed doors which opened out horizontally and closed at an angle (e.g., Figure 4b), the Lunigiana two‐door traps were separate pieces which fit into vertical slats at trap entrances and fell vertically. The one‐door traps used in Dublin for the subsequent capture seasons also had vertically closing doors (e.g., Figure 4d).

Eleven main types of bait were used and in high quantities to incentivise foxes to enter traps. Typically one type of bait was added at a time, but as traps were not cleaned between baiting, multiple bait types could be present. Bait was replaced everyday while trapping efforts were active. Baits used in Dublin included chicken, whole pigeon carcasses, household food waste, venison and tinned sardines. In Lunigiana, chicken bones and liver, roe deer meat, hare scraps, dry dog food and wet cat food were used as bait. There were no consistent differences in baits used at different trapping sites. Small amounts of bait items were spread around the trap so that foxes could sample the food before entering the trap, thought to further incentivise wild animals to enter traps.

### 2.4 Animal Handling

Captures of non‐target species caught as bycatch were documented and released without manipulation. Captured foxes were anaesthetised by the vets authorised by the research permits in both study sites to reduce stress and risk of injury to both animals and handlers. Vets carried pentobarbital sodium in a lock box so that euthanasia could be administered in cases of capturing a fox deemed ill or injured beyond recovery, in compliance with our ethics approval. As soon as researchers arrived at the trapping site, fox body mass was visually estimated to determine drug dosages and foxes were immobilised. Injecting foxes involved the use of a custom‐made padded wall attached to a pole, which was used to restrain the fox to the end of the trap. When this was not effective, a snare loop was used to restrain the fox by the trap entrance to allow the vet to administer the drug. The fox was then left inside the trap as the anaesthetic took effect, with the research team waiting out of sight to reduce disturbance. Restraining foxes to administer anaesthetic lasted less than 1 min.

In Dublin, one of two anaesthetic drug combinations was used, as part of a separate study on anaesthesia recovery in foxes: either 0.07 mg/kg medetomidine and 0.8 mg/kg midazolam or 3 mg/kg alfaxalone and 0.8 mg/kg midazolam. Anaesthesia was reversed with atipamezole and/or flumazenil, respectively (Romero Marco et al. 2025). In Lunigiana, foxes were anaesthetised using 4 mg/kg tiletamine‐zolazepam and 0.08 mg/kg medetomidine and reversed with atipamezole. In all cases, the anaesthetic was administered intramuscularly in the hind‐quarter by handheld injection. The vet assessed the sedated fox for visible signs of injury or ill health, based on overall appearance and including checking the teeth and gums. Sex and body mass were recorded for all individuals, and females were palpated to check for pregnancy. Age category was simply recorded as adult or juvenile, as it is difficult to visually assess fox age after the first 6 months (Harding et al. 2001), and trapping efforts did not coincide with the cub season. In Dublin, heart rate, respiratory rate, body temperature, haemoglobin oxygen saturation levels and blood pressure were additionally monitored during anaesthesia, and blood samples were taken as part of the anaesthesia study (Romero Marco et al. 2025). Blankets and/or bubble wrap were used to maintain body temperature as required. Foxes were placed back in traps after the anaesthetic reversal agent had been administered and released when they were alert and witnessed moving in the trap with no visible ataxia. Foxes were observed upon release until out of sight.

### 2.5 Collar Deployment and Retrieval

**FIGURE 5:**
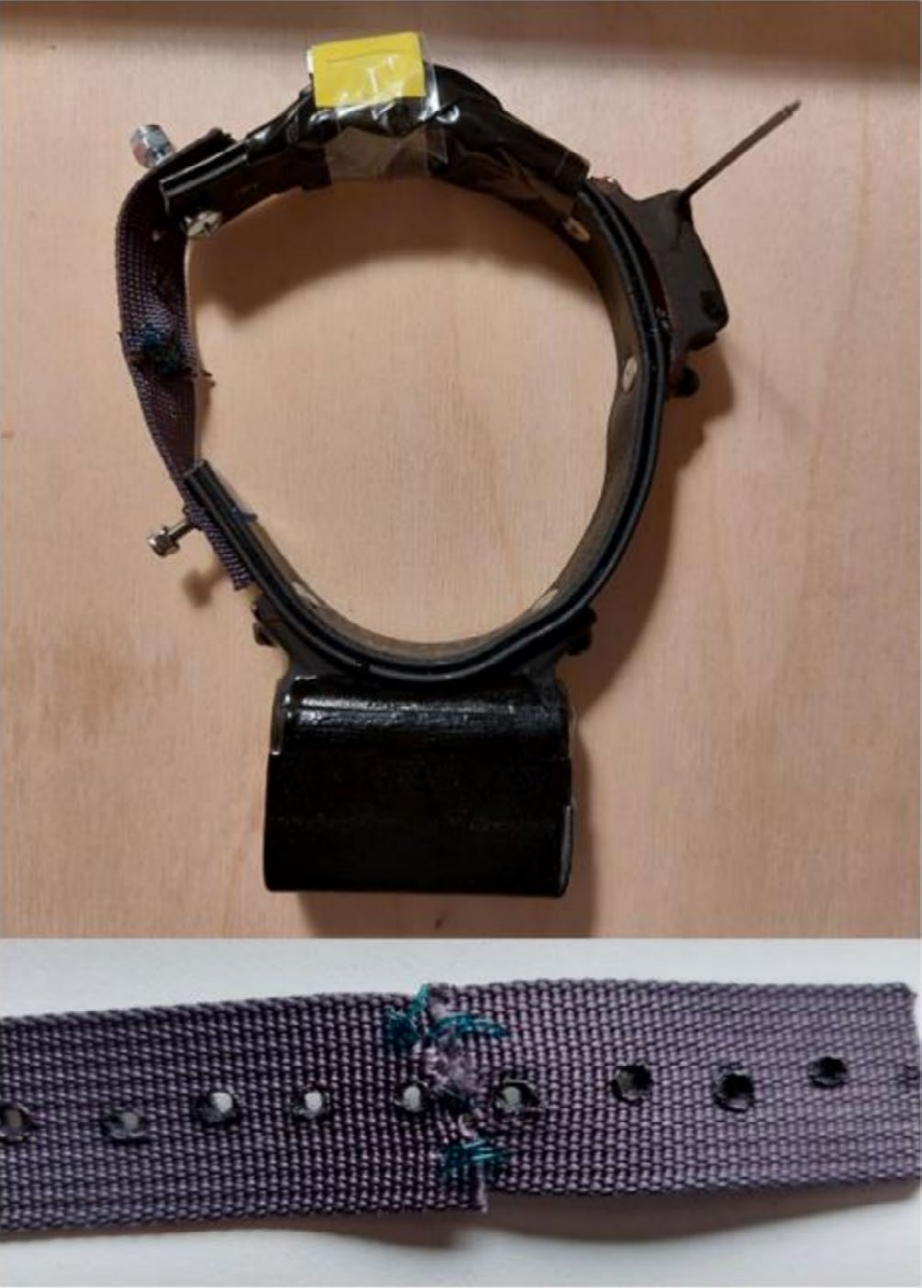
Collars were modified to include a suture‐based drop‐off mechanism. The leather collars were cut and sewn back together with webbing and absorbable surgical suture (see inset), which degrades over time with exposure to moisture. Note that the yellow tape on top of the collar is securing a magnet which is removed from the VHF unit prior to collar deployment. The collar shown here is the model including the Daily Diary compartment.

We used Axytrek collars (Technosmart, Italy) to collect GPS locations. UHF remote transmission and download was possible using an associated mobile base station, a small waterproof unit with a solar panel. The base station was placed in park depot capture sites or nearby gardens with known fox visitation for up to 1 week at a time. In Dublin, 13 collars were also equipped with a Daily Diary biologger (hereafter DD; Wildbyte Technologies, UK; Wilson et al. 2008). The DD and an AA cell Saft battery were in a separate compartment added to the base of the custom‐made collars (Technosmart, Italy; Figure 5). Attaching the DD in this way kept the collar more streamlined and helped minimise the overall weight of the collar. Collar mass was 90 g without the DD and associated battery, or 120 g including these. All collars were further equipped with a lightweight VHF unit for monitoring and collar retrieval. All VHF units used in 2023 were from Advanced Telemetry Systems (USA). In 2024, lost units were replaced with new units from Perdix (UK). VHF units were glued to the collar strap using epoxy resin and further secured with small cable ties, with the ends clipped and melted to ensure no sharp edges, and an outer layer of duct tape (Figure 5).

We made collars with degradable drop‐off mechanisms to allow collar recovery and for welfare purposes, to ensure collars would not stay on foxes indefinitely. We considered two key potential consequences of automated, electronic drop‐offs. The first is the risk that the mechanism would fail, which can commonly occur with species with underground dens, where the drop‐off mechanism may become clogged with soil (Matthews et al. 2013). The second consideration is added weight to the collar, which is particularly important to consider for smaller animals (Matthews et al. 2013; Rafiq et al. 2019). Drop‐off mechanism weight is significant when trying to stick to the standard rule of thumb of keeping tag weight < 3% of the tagged animal's overall body mass (Kenward 2000). Collars were not to be deployed on any foxes weighing < 4 kg (i.e., in which case the collar would surpass 3% of body mass); however, all captured foxes surpassed this minimum weight threshold.

To construct the degradable drop‐off mechanisms, we cut the leather collar and added a weaker material section repurposed from rucksack strap webbing. The webbing section was added where the collar was fastened, and so holes were added to facilitate collar fit with either a soldering iron or handheld hole punch tool. This section was further cut in two and sewn back together with surgical suture (Figure 5). Three suture types were used with different manufacturer‐specified resistance and absorption rates (Table 2). Polyglactin 910 was used for all drop‐off mechanisms in Dublin (*n* = 15), while Polydioxanone (*n* = 2) and Poliglecaprone 25 (*n* = 1) were used in Lunigiana. Critically, all suture types were absorbable, meaning they degrade over time with exposure to moisture as well as wear. Approximate durations of collar wear before drop‐off breakage were estimated for collars with known fates based on the collar data itself, camera trap footage, public sightings and VHF‐confirmed movement. All collars were labelled with researcher and university names as well as contact information so that members of the public could report dropped collars. This information was hand‐written in 2023, while label‐maker printed labels were used in 2024. Both labelling methods were covered with an epoxy resin layer to minimise weathering. The field team was not able to monitor collar fates closely in 2024; as such, collar deployment durations were estimated for 2022 and 2023 only.

**TABLE 2:** Drop‐off specifications and approximate duration of each suture drop‐off per confirmed dropped collar with known attachment length.

Year	Location	Collar ID	Suture material	Tensile resistance	Total absorption	Approx. drop‐off duration
2022	Lunigiana	162	Polydioxanone	42 days	182–238 days	29 days^a^
		182				65 days^b^
2023	Dublin	51	Polyglactin 910	32 days	56–70 days	154 days
		53				3 days
		54				211 days
		55				52 days
		59				36 days^c^

*Note:* Tensile resistance and total absorption are according to manufacturer guidelines for the material's intended purpose to degrade inside the body following surgery. Tensile resistance is the timeframe after which the material loses its strength, whereas total absorption is when the material has completely dissolved in the body cavity through hydrolysis. These timeframes are not expected to be accurate for collar‐attached suture but may provide a guideline when considering suture‐based drop‐off mechanisms. Note that no data are available for the Poliglecaprone 25 suture type, which was used for one collar that was not recovered.

^a^ Fox recaptured and collar swapped.

^b^ Fox shot and collar reported to local management agency.

^c^ Collar not recovered but drop confirmed from camera trap.

### 2.6 Capture Success Likelihood by Weather Conditions

Minimum and maximum daily temperature and rainfall data were obtained from the Phoenix Park weather station in Dublin for February and March 2023 and 2024 via Met Éireann, the Irish Meteorological Service (see https://www.met.ie/climate/available‐data/historical‐data). We used an asymptotic Wilcoxon–Mann–Whitney test from the *coin* package (Hothorn et al. 2006) in R 4.3.1 (R Core Team 2023) to test if weather conditions on nights traps were armed affected the likelihood of capture success. We then used the *climwin* package to further test whether these weather conditions affected capture rate over longer time periods (Bailey and van de Pol 2016), following the workflow set out by van de Pol et al. (2016). The *slidingwin* function was used to test the effect of weather conditions up to 7 days before each trapping night on capture rate. This function created a temporal sliding window, assessing each weather variable in turn, compared to a baseline intercept‐only linear model. Weather condition analyses were carried out on the Dublin data only due to the low number of fox captures in Lunigiana.

## 3 Results

In Dublin, there were three capture seasons: Autumn 2022, Spring 2023, and Spring 2024. The initial capture season in Autumn 2022 ran from the 26th of September to the 30th of November 2022 across two capture sites and did not lead to any successful fox captures. Nightly fox activity was confirmed for both sites from camera trap footage, providing confirmation of fox presence in the trapping sites. Further, camera traps showed foxes triggering traps while evading capture, for example, by stretching to reach centrally placed bait while leaving at least one paw outside the trap (Figure 4b). The decision to switch to longer traps and monitor multiple trapping sites simultaneously with remote monitoring systems was made at the close of this capture season.

Foxes were successfully captured during both of the subsequent capture seasons in February–March 2023 and 2024, respectively. Traps were set for 14 nights during the 2023 capture season, with seven nights having successful fox captures (50% success rate). Eight foxes (three females, five males) were trapped and collared between the 19th and 28th of February 2023 (Table 1). An additional fox in poor condition was captured and humanely euthanised during this time. Capture efforts briefly continued from the 13th to the 16th of March 2023, with no further captures. Captures occurred at five of the six trapping sites used in 2023.

In 2024, traps were set for 31 nights, with eight nights having successful fox captures (25.8% success rate). Seven unique foxes (four females, three males) were captured and collared in Dublin between the 8th of February and the 21st of March 2024 (Table 1). The eighth successful fox capture was a recapture of one of these seven, leading to immediate release without manipulation. Captures occurred at two of the four trapping sites used in 2024.

In total, we had 16 fox captures in Dublin over 45 trapping nights between the 2023 and 2024 seasons, giving a trapping efficiency of one fox per 2.8 trapping nights. Excluding recaptures, the sex ratio of captured foxes in Dublin from 2023 to 2024 combined was 1:1 (7 males, 7 females; Table 1).

In Lunigiana, there were three fox captures between the 8th of February 2022 and the 9th of March 2022, with one of these being a recapture (Table 1). Both individuals captured were female. Trapping efforts continued without further success until the 20th of May, when traps were closed for the breeding season. Traps were reset for an autumn capture season without any further captures. All fox captures occurred in a single trapping site, which was at the edge of a small village. This site was unlike the six other trapping sites used, which were all positioned off hiking trails in forested areas (Table 1). Camera traps positioned at each trapping site confirmed fox presence at each one. There was no consistency in bait type between these three capture events, with the traps containing a different main bait item each time. These were roe deer meat, dry dog food and meat scraps, respectively.

### 3.1 Bycatch

Bycatch was rare in Dublin, though badgers and cats were captured on two occasions each in park depots. One domestic dog of the Labrador breed investigated, took bait and triggered the trap in the private garden location on multiple instances but was too large to be trapped and showed no adverse reaction to the motion of the trap door when triggered. These bycatch captures all occurred during the night when traps were armed. In contrast, 30 individuals across seven species were caught as bycatch in Lunigiana: domestic cat (*Felis domesticus*, *n* = 14), common buzzard ( *Buteo buteo* , *n* = 6), crested porcupine ( *Hystrix cristata* , *n* = 4), beech marten ( *Martes foina* , *n* = 3), European hedgehog ( *Erinaceus europaeus* , *n* = 1), European polecat ( *Mustela putorius* , *n* = 1) and European badger ( *Meles meles* , *n* = 1). No trap‐related injuries were observed in Dublin or Lunigiana, in the target or bycatch species.

### 3.2 Data Retrieval and Suture Drop‐Offs

We recovered GPS data from two of three individuals in Lunigiana (66.66%), six of eight individuals collared in Dublin in 2023 (75%) and five of seven individuals in Dublin in 2024 (71.42%). Accelerometer data were also recovered from two of three individuals in Lunigiana (66.66%) as these were combined units. DD data were recovered from 50% of individuals in 2023.

In Dublin, four of the eight deployed collars were retrieved from the 2023 capture season; as such, the breakage of the drop‐offs was confirmed for these cases (Table 2). Further, one fox had an identifiable scar over his eye and was seen on a camera trap 36 days after collar deployment without the collar (Table 2). As such, while the collar was not recovered, the drop‐off also seems to have worked in this case. We received some reports from members of the public who had sighted collared foxes, but none later than July in 2023, supporting potential drops of unretrieved collars. Collar retrieval was made more difficult due to the limited range of both the VHF units and GPS base station. Unretrieved collars are suspected to have been disposed of by members of the public.

Of the five confirmed dropped collars in Dublin in 2023, the first three collars to drop had been worn by males and dropped 3–52 days following deployment, as deduced from the collar data for the two males for which collars were retrieved and from camera trap footage for the third male for whom the collar was not relocated. Two collars worn by females dropped off after an estimated 154 and 211 days, respectively (Table 2). Two of three deployed collars were recovered in Lunigiana but neither through drop‐off breakage (Table 2). In the first case, the fox was recaptured and in the second case the same individual had been shot by poachers. In both instances, the drop‐off showed signs of visible wear.

### 3.3 Capture Success Likelihood by Weather Conditions

**FIGURE 6:**
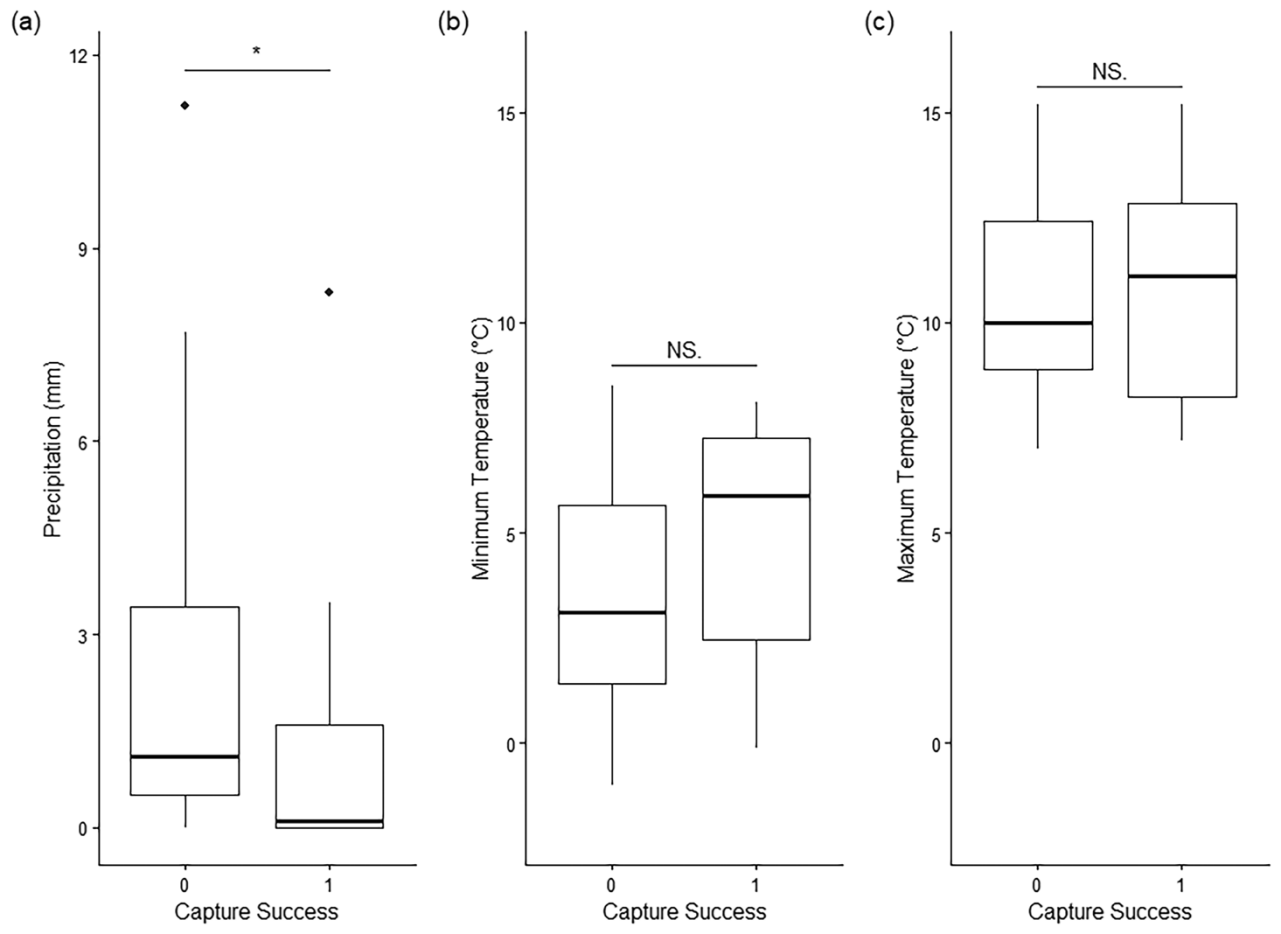
Capture success or failure in relation to (a) precipitation, (b) minimum temperature recorded on the day of trapping attempt and (c) maximum temperature recorded on the day of trapping attempt. Data are presented from 45 trapping nights in Dublin. The asterisk indicates the significant relationship detected between capture success and precipitation.

Neither minimum daily temperature (*n* = 45 trapping nights, *Z* = −1.42, *p* = 0.16) nor maximum daily temperature (*n* = 45 trapping nights, *Z* = −0.17, *p* = 0.87) on the date of capture affected capture success. Capture success had a significant relationship with precipitation (*n* = 45 trapping nights, *Z* = 2.17, *p* = 0.03) on the date of capture, with more fox captures occurring on nights with lower rainfall (Figure 6). Sliding window analysis found no significant relationships between capture rate and precipitation or temperature in the week preceding trapping (*n* = 42 trapping nights). The best fit model found a negative relationship between precipitation and capture rate using a temporal window of 0–6 days prior to the trapping night, though this was not statistically significant (*β* = −0.019, SE = 0.01, *t* = −1.86, *p* = 0.071, *R* ^2^ = 0.079). The best fit models for minimum daily temperature (*β* = −0.014, SE = 0.008, *t* = 1.54, *p* = 0.131, *R* ^2^ = 0.056) and maximum daily temperature (*β* = −0.007, SE = 0.01, *t* = −0.72, *p* = 0.47, *R* ^2^ = 0.013) were also not significant.

## 4 Discussion

Here, we report our strategies to refine fox capture and data retrieval efforts over 3 years of trapping in two distinct settings. A checklist of recommendations is provided in Box 1. Briefly, we found improved trapping success when using larger traps and operating multiple trapping sites simultaneously, with remote camera traps and trap alert tags being favoured over more frequent manual trap checks (Figure 1).

Capture success is often expressed as the number of fox captures per number of trapping nights. Our trapping efficiency of one fox per 2.8 trapping nights in Dublin is quite high compared to other studies. The capture efficiency for Lunigiana was lower than Dublin, but still favourable compared to previous studies with 3 captures in the initial capture season of an estimated 122 trapping nights, or one fox per 40.66 trapping nights. Subsequent capture efforts with no fox captures followed, however. Kay et al. (2000) report a trapping efficiency of one fox per 147 trapping nights, Muñoz‐Igualada et al. (2008) report 18 foxes per 1000 trapping nights (i.e., one fox per 55 trapping nights) and Main (2020) reports four foxes in 96 trapping nights (i.e., one fox per 24 trapping nights). Notably, the most efficient of these, Main (2020), was also conducted on an urban rather than rural fox population. Urban foxes have previously been shown to be bolder than rural foxes in approaching novel food‐related objects, though they were not more innovative in exploiting these food sources (Blake Morton et al. 2023). Further, fox population densities are often reported as being higher in urban settings compared with more natural environments (Delcourt et al. 2022), which may affect capture success through differences in the number of unique individuals encountering traps. Capture success may also vary seasonally, though previous work on another urban population did not find seasonal differences in the capture rates of adult foxes (Baker et al. 2001).

While only females were captured in Lunigiana, this sample size is too low to make meaningful inferences about sex ratios of captured animals in the region. An equal number of unique males and females were captured in Dublin, though both recaptures were male. The sex ratio of trapped foxes seems to vary considerably, with previous studies reporting no sex differences in trappability (Baker et al. 2001), bias towards male captures (Kay et al. 2000) and bias towards female captures (Henry et al. 2005), respectively. As such, sex differences may be important to evaluate and document for each population.

Cage traps are commonly used for animal capture due to their low risk of significant injuries (Iossa et al. 2007; McCarthy et al. 2013). Foxes and other species can be reluctant to enter cage traps, however; this is likely why it was important here to arm multiple traps simultaneously to increase capture success. Indeed, previous studies report improved capture success when setting multiple traps (Kay et al. 2000; Kamler et al. 2002). Trap length was also important to consider. While the traps used in the initial 2022 capture season in Dublin were advertised specifically as fox traps, they were found to be too short to ensure the capture of the foxes that entered these traps. Ensuring appropriate trap measurements may be especially important when purchasing traps that are manufactured elsewhere and imported, as foxes range in body size across their geographic distribution (Cavallini 1995). For this reason, we recommend checking trap measurements of advertised traps against trap measurements reported in successful capture studies of the target species instead of relying upon manufacturer guidelines. Potential differences in body size between the target study area and reference studies should also be considered.

A need to continuously evaluate and refine trapping methods has been identified (Iossa et al. 2007; Muñoz‐Igualada et al. 2008; Caravaggi et al. 2021). Specifically, injury rates and trap selectivity (i.e., prevalence of bycatch) have been highlighted as requiring increased consideration and reporting (Iossa et al. 2007; White et al. 2021). The most frequently reported injuries associated with cage traps tend to be tooth damage, associated with animals chewing on the wire mesh of the trap, and skin abrasions, typically to the limbs or snout (Woodroffe et al. 2005; McCarthy et al. 2013; White et al. 2021). Here we had no instances of such injuries. While bycatch captures were released without the sedation and full veterinary assessment received by captured foxes, no visible injuries were observed in bycatch species. There was a high occurrence of non‐target species bycatch in Lunigiana compared to Dublin, with 30 of 33 capture events in Lunigiana capturing non‐target species (91%). High bycatch may have affected capture success as an occupied trap could not capture foxes. Differences in bycatch capture rates are likely due to Lunigiana being a wilder, more biodiverse study site. High levels of bycatch associated with cage traps have been reported in other studies (Shivik et al. 2005; Muñoz‐Igualada et al. 2008), though their low reported injury rates warrant continued use.

One possible refinement to minimise bycatch may include testing for more target‐species specific baits or lures (Shivik and Gruver 2002; McCarthy et al. 2013). While live bait and the use of fox urine as a lure have been found to increase red fox capture efficiency, neither led to a reduction in bycatch (Díaz‐Ruiz et al. 2016), and using live bait has separate welfare concerns for the animals being used as bait (Caravaggi et al. 2021). Lures may warrant further consideration; however, as canid‐specific lures have shown promise in improving trapping selectivity elsewhere (Shivik and Gruver 2002). In the current study, capture success varied considerably by trapping site (Table 1), and we suspect the scent of previously captured conspecifics may have acted as an attractant, as has been documented in other species (Pawlina and Proulx 1999; Martin et al. 2014). As such, scent‐based baits may be worth exploring in future trapping efforts.

Of the seven trapping sites used in Lunigiana, only one had successful fox captures. Notably, this capture site was on the edge of a small village, unlike all the others which were in forested areas (Table 1). We suspect that novel objects such as traps seem less daunting close to human settlements where the presence of novel human objects is high, compared with natural woodland areas. Based on our experience in Lunigiana, we expected that urban foxes would be less wary of traps due to familiarity with human activity and scent. Urban animals also encounter novel human‐made objects frequently, but urban foxes were also wary around traps (Figures 3 and 4). Urban wildlife populations can show reduced neophobia and higher tolerance of novel objects to exploit feeding resources (Lefebvre 1995; Lowry et al. 2013). However, urban wildlife can also be at increased risk of primary and secondary poisoning (Riley et al. 2014). As such, urban wildlife can also exhibit high neophobia to avoid persecution, as has been documented in fox populations elsewhere (Padovani et al. 2021). The degree of neophobia may be object‐specific, but with urban populations being faster to explore novel objects than rural counterparts (Greggor et al. 2016). Habituation periods for traps set in urban environments are valuable in the context of urban fox neophobia, and the habituation process may be faster in human‐dominated environments compared to adjacent natural areas.

Our ability to monitor multiple trapping sites without extending the amount of time captured foxes spent in traps was facilitated by the dual use of trap alert tags and remote transmission camera traps. Trap alert tags notified the capture team of any trap closures, while remote transmission camera traps allowed verification of capture as well as identifying the captured species. The use of these technologies in tandem also ensured there was a back‐up if either system failed, which occurred once with the remote cameras in the 2024 capture season. Manual trap checks were continued once per day, which also served as an opportunity to top up bait as necessary, but these technological aids ensured faster, prioritised arrivals at traps where a capture had taken place. Remote monitoring of trapping sites also minimised disturbance, which may improve capture success of trap‐shy species (Arthur 1988; McCarthy et al. 2013). Further, remote monitoring conserved researcher effort, improved coordination and minimised fatigue, making trapping periods more sustainable and reducing the chance of tiredness‐induced accidents in the field (Campbell and Griffith 2015; Keiter et al. 2022). False triggers or bycatch captures can be dealt with by one team member while full capture teams can immediately coordinate arrival for target species captures. While cellular signal can be inconsistent in some study areas (Keiter et al. 2022), these remote monitoring tools are valuable in urban areas with reliable coverage. Additional considerations may also be required in study areas along international borders. Such tools are likely to be viable across larger areas as satellite communication systems become more affordable.

Camera traps contributed considerably to our understanding of fox behaviour around traps and potential reasons for unsuccessful trapping attempts. Traps were armed only when foxes were confirmed to be both visiting the trapping sites and entering traps (Figure 3), which conserved the researchers' effort required in monitoring active traps. It was through camera trap footage that we realised our first traps were too short in length and had doors that could be pushed up by foxes, despite being advertised as fox‐specific traps (Figure 4). Fox capture rates have been found to decrease with successful captures in neighbouring traps for longer trapping periods (Ruette et al. 2003), which may indicate captured fox reluctance to re‐enter traps but also potentially learned trap avoidance from conspecifics. Trap aversion has been shown in captive coyotes, for example, which socially learn trap avoidance behaviour from demonstrators (Young et al. 2022).

Collar failure rates can be high, significantly impacting sample sizes in movement ecology (Hebblewhite and Haydon 2010). For example, Hofman et al. (2019) reported that 48% of GPS collar deployments end prematurely and many studies report significant proportions of deployed collars undergoing technical failure and/or retrieval failure (Gau et al. 2004; Kaczensky et al. 2010; Matthews et al. 2013; Jung et al. 2018; Studd et al. 2021). These high failure rates can be particularly detrimental when working with difficult‐to‐capture species. Here, we successfully recovered data from all retrieved units and recovered additional data from unretrieved collars through base station download. Remote transmission through the base station significantly boosted data retrieval, with two of six GPS datasets recovered in 2023 being through the base station alone and all 2024 GPS data collected obtained via the base station. This is despite the base station range being significantly more limited than expected, as ascertained from the base station failing to connect to collars with collared fox presence confirmed via VHF tracking. Limited base station range may have biased the collected data by failing to connect to the collars of wider‐ranging or dispersing foxes. More detailed reporting of collar specifications and data recovery may be particularly important in urban areas, where data transmission capabilities are not as well understood as other environments (Adams et al. 2013). Another study on urban foxes collected data from five individuals, with four of these five only connecting to the base station once and the other three times (Kobryn et al. 2023). As such, there is a clear need for further testing of VHF and GPS unit transmission strength and range in urban environments. We recommend thorough testing to establish these parameters in study areas ahead of collar deployment, rather than reliance on manufacturer guidelines. Collars which remotely transmit data via satellites or the Global System for Mobile communications (GSM) may resolve this issue in the future, though they come with increased costs (Pastorini et al. 2015). Long Range Wide Area Networks (LoRaWAN), which involve the use of a system of gateways, show considerable promise as a low‐cost data transmission option (Gauld et al. 2023). More simply, base stations which record the times of data download may help to refine programmed remote download intervals in the future.

Suture drop‐offs can act as a lightweight alternative to electronic drop‐off systems. Suture may also be cost‐effective, particularly where expired suture not fit for surgical use can be repurposed, as was the case here. We report five estimated drop‐off durations from the Dublin 2023 capture season (Table 2). Unrecovered collars likely either dropped in inaccessible locations such as dens or were discarded by members of the public. The risk of disposal may be high in areas of dense human population, even where collars are labelled, as members of the public may not inspect these closely. For confirmed dropped collars, there was considerable variation in the duration each drop‐off lasted, which may result from individual variation in force exerted on the collar. Of the five collars with drop‐off estimates, the collars with the three shortest confirmed drop‐off durations were worn by males and the two longest were worn by females (Table 2). These preliminary data suggest a difference in force exerted upon collars between males and females, warranting future consideration with larger sample sizes. Using a stronger drop‐off mechanism to increase data collection durations can help ensure maximum information gain following the capture event, though care should always be taken to ensure the drop‐off will break as intended. We also suggest researchers experiment with different types of suture with different tensile resistance and absorption specifications.

Animal responses to weather conditions can affect activity levels and therefore the likelihood of trap visitation and capture success (Cox and Hunt 1992; Pawlina and Proulx 1999). Minimum and maximum recorded temperatures on days where traps were set did not affect capture success. Precipitation on the date of capture did have a significant relationship with capture success, with capture likelihood decreasing with increased rainfall. Foxes in Dublin may be less active on nights with higher rainfall and therefore less likely to enter traps, explaining why 2023 had a greater proportion of successful trapping nights than 2024. March 2023 and 2024 were two of the wettest on record in Ireland. While February 2024 also had high rainfall, February 2023 was drier (Met Éireann 2024; RTÉ News 2024) and all 2023 captures occurred in February. Temperature may be more important in other seasons, particularly in regions experiencing both higher and lower temperature extremes than typical in Ireland's mild climate. Further analysis did not find a significant relationship between capture success and precipitation or temperature conditions in the week prior to each trapping night, though this may be worth further consideration at larger sample sizes (van de Pol et al. 2016). There are contrasting reports of which seasons are associated with higher trapping success for foxes (Baker et al. 2001; Valcarcel et al. 2020), indicating that this may vary with local climate conditions.

BOX 1 **Checklist of recommendations for capturing wild foxes.**

1. Choose safe capture locations where the risk of disturbance from members of the public is minimal.
2. Choose a time interval for trap checks which optimises the welfare of study animals while remaining mindful of practical considerations.
3. Decide how many traps to arm simultaneously and how many trapping sites to use, determined by the length of time required to process the animal upon capture and travel time between trapping sites.
4. Ensure all traps are of adequate length to trap foxes, relying on previous studies over manufacturer guidelines and accounting for geographic variation in fox body size.
5. Choose a pre‐baiting timeframe that allows target animals to become familiar with traps, informed by fox behaviour observed on camera traps.
6. Test the signal strength and range of transmitting VHF and GPS units in advance of deployment.
7. Include a drop‐off mechanism to account for low recapture rates of collars.
8. Evaluate whether local weather conditions are influencing trap visitation and therefore capture success.

## Author Contributions

**Holly M. English:** conceptualization (equal), data curation (lead), formal analysis (lead), funding acquisition (supporting), investigation (equal), methodology (equal), writing – original draft (lead), writing – review and editing (equal). **Patricia Romero:** investigation (equal), methodology (equal), writing – review and editing (equal). **Lorraine Bull:** funding acquisition (equal), project administration (equal), resources (equal), writing – review and editing (equal). **Barry Nolan:** methodology (equal), resources (equal), writing – review and editing (equal). **Paolo Bongi:** data curation (supporting), funding acquisition (equal), investigation (equal), methodology (equal), resources (equal), writing – review and editing (equal). **Vilhelmiina Huuskonen:** resources (equal), supervision (supporting), writing – review and editing (equal). **Simone Ciuti:** conceptualization (equal), funding acquisition (equal), investigation (equal), methodology (equal), project administration (equal), supervision (lead), writing – review and editing (equal).

## Conflicts of Interest

The authors declare no conflicts of interest.

## Data Availability

Data from weather stations is open access and available from Met Éireann at https://www.met.ie/climate/available‐data/historical‐data. Data on capture success by trapping date is available at https://github.com/hollyenglish/fox_capture.
